# Characterization of normal and cancer stem-like cell populations in murine lingual epithelial organoids using single-cell RNA sequencing

**DOI:** 10.1038/s41598-021-01783-5

**Published:** 2021-11-16

**Authors:** Erik Johansson, Hiroo Ueno

**Affiliations:** 1 Department of Stem Cell Pathology, Kansai Medical University, 2-5-1 Shin-machi, Hirakata, Osaka 573-1010 Japan; 2 CREST, Japan Science and Technology Agency, 4-1-8 Honcho, Kawaguchi, Saitama 332-0012 Japan

**Keywords:** Cancer, Oral cancer, Cell growth

## Abstract

The advances in oral cancer research and therapies have not improved the prognosis of patients with tongue cancer. The poor treatment response of tongue cancer may be attributed to the presence of heterogeneous tumor cells exhibiting stem cell characteristics. Therefore, there is a need to develop effective molecular-targeted therapies based on the specific gene expression profiles of these cancer stem-like cell populations. In this study, the characteristics of normal and cancerous organoids, which are convenient tools for screening anti-cancer drugs, were analyzed comparatively. As organoids are generally generated by single progenitors, they enable the exclusion of normal cell contamination from the analyses. Single-cell RNA sequencing analysis revealed that p53 signaling activation and negative regulation of cell cycle were enriched characteristics in normal stem-like cells whereas hypoxia-related pathways, such as HIF-1 signaling and glycolysis, were upregulated in cancer stem-like cells. The findings of this study improved our understanding of the common features of heterogeneous cell populations with stem cell properties in tongue cancers, that are different from those of normal stem cell populations; this will enable the development of novel molecular-targeted therapies for tongue cancer.

## Introduction

Squamous cell carcinoma of the tongue is the most common malignancy of the oral cavity^1^. The incidence of oral cancer in young patients is steadily increasing, independent of traditional risk factors such as tobacco and alcohol use. This increase is due to the rise of tongue and oropharyngeal carcinomas, in the latter case related to human papilloma virus (HPV) infection^2^. The prognosis of young patients is heterogeneous. However, several studies have demonstrated an increased risk of relapse and a trend of poor prognoses in this cohort. The poor outcome and resistance to treatment may be due to the presence of genetically heterogeneous populations of cells with stem-like features in the tumors^3^. However, effective molecular-targeted therapies for tongue cancers based on the genetic profiles of cancer stem-like populations have not been developed. To improve the prognosis of tongue cancers, the differential characteristics of cell populations with stem cell properties in healthy and malignant tongue epithelial tissues must be elucidated.

Several methods have recently been established for the three-dimensional (3D) culture of stem cells isolated directly from various tissues^4,5^. These 3D structures are termed “organoids” because they resemble the organ from which they are isolated in terms of cellular composition, gene expression, and function—even after long-term expansion. Organoids can be used to recapitulate organ development and maintenance, examine rare stem cell populations that are difficult to monitor in vivo, and model diseases. Cancer organoids are particularly convenient tools for screening anti-cancer drugs. We previously identified mouse lingual epithelial stem cells^6^ and established organoid cultures thereof^5^. Additionally, candidate stem-like cells in tongue cancer tissues were identified in mice^1^.

This study examined the effects of 4-nitroquinoline-1-oxide (4-NQO), a commonly used carcinogen to induce tongue cancer in mice that mimics the effects of tobacco use by promoting the formation of DNA adducts^1,7^. Previous studies have demonstrated that the tumors that are formed histologically and genetically mimic human squamous cell carcinomas of the tongue^8,9^. Additionally, previous studies on the mutational landscape of 4-NQO-induced tumors revealed that common mutations in human tongue carcinomas, including *Trp53*, *Pik3ca*, and *Notch1*, were recapitulated in the mouse model^10^. Therefore, this model was selected for this study.

This study aimed to characterize cells with stem cell-like features in normal and cancerous tongue epithelial tissues. To enrich rare stem cell populations, we isolated cells from lingual epithelium of untreated and 4-NQO-treated mice and expanded progenitor cells in organoid cultures in vitro. The findings of this study demonstrated that cancer organoids mimicked the histological features of lingual cancer tissues. Single-cell RNA sequencing (scRNA-seq) analysis revealed that negative regulation of the cell cycle and the activation of p53 signaling were enriched characteristics of stem-like cells from normal organoids whereas hypoxia-related pathways, such as HIF-1 signaling and glycolysis, were upregulated in stem-like cells from cancer organoids.

## Results

### Induction of tongue cancer

**Figure 1:**
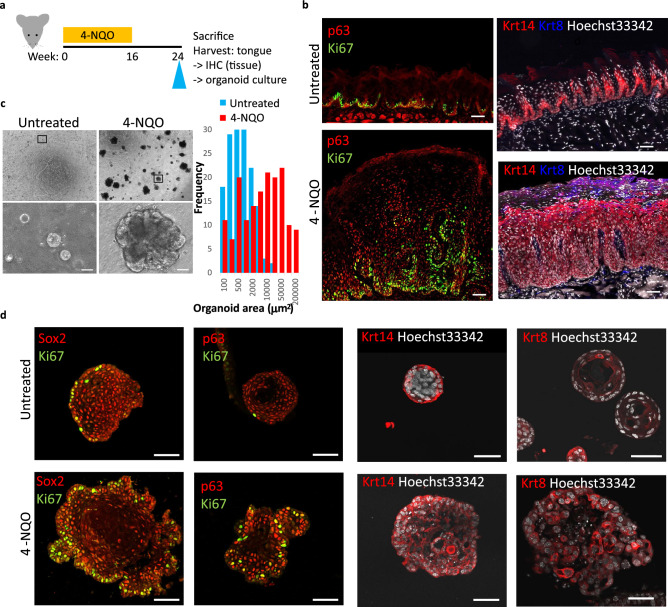
Induction of tongue cancer and histological analysis of non-malignant and malignant lingual epithelial tissues and organoids. (**a**) Scheme for 4-nitroquinoline-1-oxide (4-NQO) treatment of mice and tongue harvesting. (**b**) Immunostaining of lingual epithelial tissue of untreated (upper panels) and 4-NQO-treated (lower panel) mice with the indicated primary antibodies. (**c**) Isolated lingual epithelial organoids from untreated (left) and 4-NQO-treated (right) mice at day 12 after single-cell seeding in Matrigel. The lower panel shows the magnified images of the upper panel. The graph shows the frequency of organoids of different sizes from untreated and 4-NQO-treated mice. (**d**) Immunostaining of lingual epithelial organoids from untreated (upper panel) and 4-NQO-treated (lower panel) mice with the indicated primary antibodies. Scale bars = 50 µm.

To induce tongue cancer, Sox2-GFP mice were administered 4-NQO (100 µg/mL) through drinking water for 16 weeks. Next, the mice were administered plain drinking water for 8 weeks and sacrificed. The tongue was harvested and subjected to immunohistochemical analysis and organoid culture (Fig. 1a). The lingual epithelium of untreated mice was covered with cone-shaped filiform papillae. The expression of Krt14, p63, and Ki-67 was detected in the basal layer of the epithelium of untreated mice. In contrast, 4-NQO-treated mice exhibited thick focal lesions with disrupted epithelial organization lacking filiform papillae. The expanded basal layer exhibited widespread expression of Krt14, p63, and Ki-67, while the outer keratinized layers exhibited the expression of Krt8 (Fig. 1b).

### Establishment and characterization of control and cancer lingual organoids

Sox2+ keratinocytes residing in the basal layer of the tongue epithelium include stem cells for both taste and non-taste lingual epithelia^11^. Furthermore, only the Sox2+ cells formed lingual organoids in vitro. To culture normal and cancerous lingual organoids, the tongue epithelia from the control (untreated) and 4-NQO-treated Sox2-GFP mice were digested and separated into single-cell suspensions. GFP^+^ cells were isolated from the cell suspensions using a cell sorter and seeded in Matrigel for 3D organoid culture. The control organoids derived from normal tissue were round and symmetrical, whereas the 3D cultures of cells isolated from cancer tissue were characterized by large organoids that exhibited accelerated, aberrant growth and irregular shapes with bud-like proliferative structures, although some smaller organoids, similar to those from normal tissue, were formed as well (Fig. 1c). The expression of the proliferation marker Ki-67 in the cells of the outer and basal layers in cancer organoids was higher than that in the control organoids (Fig. 1d). The expression of Krt14, however, was observed only in the basal layer of control organoids but detected in the inner suprabasal layers of cancer organoids. Krt8 was widely expressed in cancer organoids but detected only in some suprabasal cells in the control organoids (Fig. 1c). These expression patterns recapitulated the immunohistochemical patterns of tongue epithelial tissue in control and cancer mice. Thus, cancer organoids can be used as an in vitro model for tongue cancer (Fig. 1b).

### scRNA-seq of control and cancer lingual organoids

**Figure 2:**
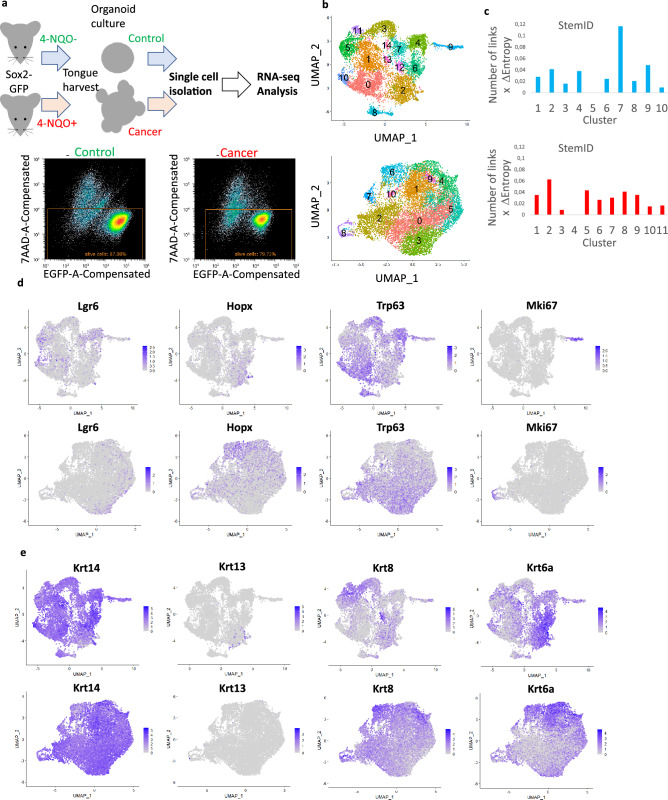
Single-cell RNA sequencing analysis of control and cancer organoids. (**a**) Scheme for single-cell isolation and RNA sequencing analysis of control and cancer lingual epithelial organoids from untreated and 4-nitroquinoline-1-oxide (4-NQO)-treated mice. The plots for sorting of alive cells for single-cell RNA sequencing. (**b**) Uniform manifold approximation and projection (UMAP) plots of control (upper panel) and cancer (lower panel) organoid Seurat datasets labeled by Seurat clusters. (**c**) Bar charts of StemID values for RaceID clusters in control (upper) and cancer (lower) organoid datasets. (**d**) UMAP plots showing the expression of epithelial stem cell and proliferation markers in control (upper panel) and cancer (lower panel) Seurat objects. (**e**) UMAP plots showing the expression of keratin genes in control (upper panel) and cancer (lower panel) Seurat objects.

To analyze the gene expression profiles between control and cancer organoids in a comparative manner, organoids derived from one control mouse and one 4-NQO treated mouse were digested and separated into single-cell suspensions. The cell suspensions were subjected to cell sorting and scRNA-seq. Approximately 100,000 cells (corresponding to approximately 500 organoids) from the control sample and 60,000 cells (corresponding to approximately 150 organoids) from the cancer sample were collected and used for subsequent scRNA-seq analysis (Fig. 2a).

### Clustering and StemID value determination for control and cancer datasets

Seurat was used to cluster and analyze control and cancer organoid datasets. The analysis revealed 14 and 10 clusters in the former and latter, respectively (Fig. 2b). To identify stem cells in the two datasets, the RaceID and FateID R packages were used to identify the clusters with the highest StemID values (most stem-like cell clusters). By comparing expression of the top markers for the RaceID clusters with high StemID values and their expression pattern in the Seurat object, the Seurat clusters enriched in stem-like cells could be determined (Supplementary Fig. 1). Interestingly, while the control organoids had one cluster (RaceID cluster 7, corresponding to Seurat cluster 5) with a significantly higher StemID score than the other clusters, none of the cancer organoid clusters had such a high score, with the highest StemID score for RaceID cluster 2 (corresponding to Seurat cluster 3), but with several other clusters displaying only slightly lower scores (Fig. 2c; Supplementary Fig. 1A). This suggests the presence of several cell populations with stem cell features in the cancer organoids, thus indicating the heterogeneous nature of tongue tumors.

### Marker expression in the lingual organoids

The expression of known basal and progenitor cell markers of the lingual epithelium in the uniform manifold approximation and projection (UMAP) plots of the Seurat objects was examined. The progenitor marker Lgr6 was expressed in the stem-like cell clusters of both control and cancer organoids, whereas Hopx was mostly expressed in clusters with low StemID values (Fig. 2d; Supplementary Fig. 1C). The number of Lgr6^+^ cells in the control organoids was higher than that in the cancer organoids, while the number of Hopx^+^ cells in the cancer organoids was higher than that in the control organoids. Mki67^+^ cells, which divide actively, were separated in a single cluster in both datasets (clusters 9 and 8 for the control and cancer organoids, respectively). The basal cell marker Krt14 was widely expressed in both control and cancer organoids. Krt13, a marker for differentiated suprabasal cells in the lingual epithelium, was expressed in cluster 2 in the control organoids but sparsely expressed in the cancer organoids (Fig. 2e). Krt8-expressing cells were observed in most cancer clusters but were few in the stem-like cell cluster. In the control, on the other hand, Krt8 was mostly expressed in the stem-like cell cluster.

### Trajectory analysis

**Figure 3:**
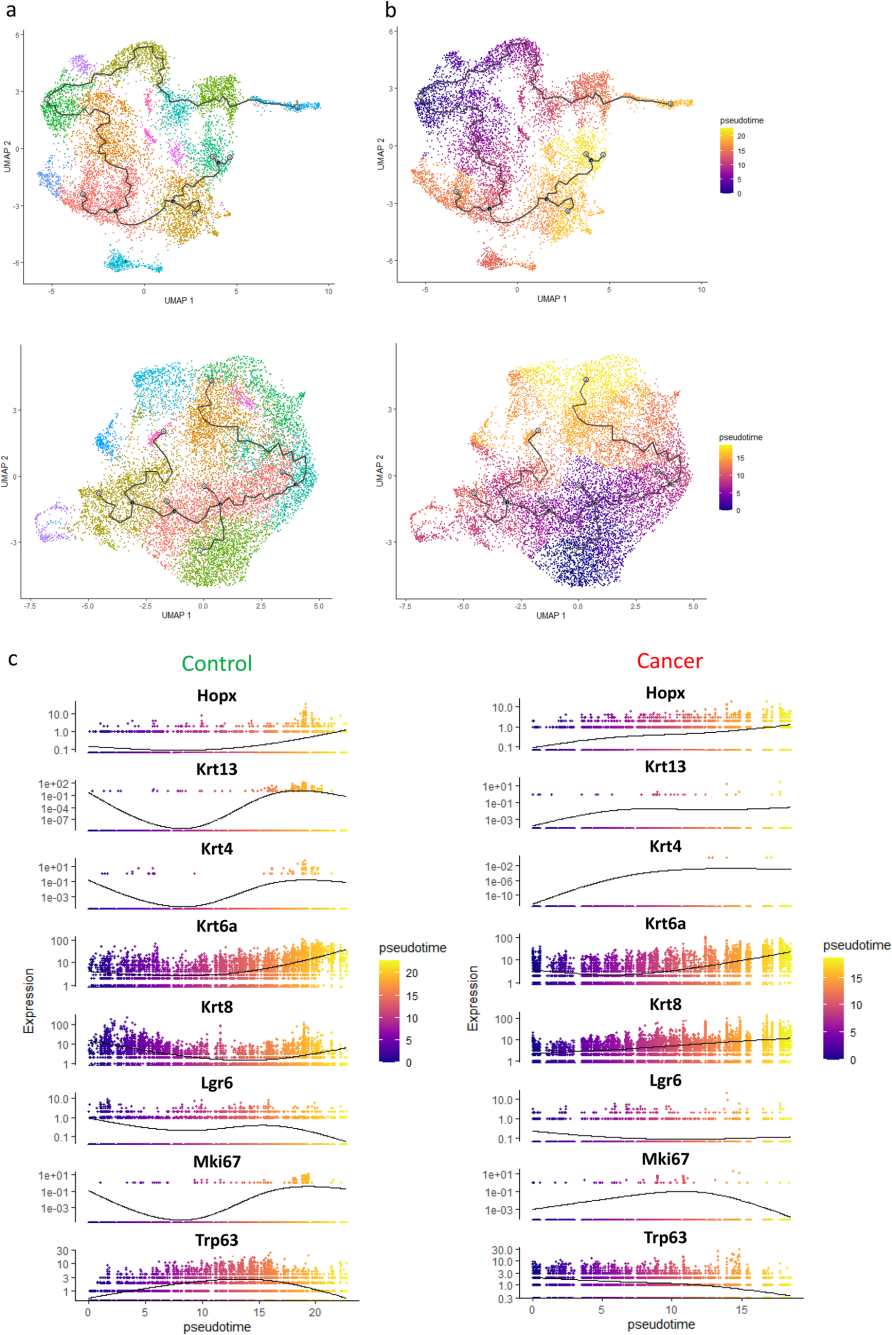
Trajectory analysis of cells from control and cancer organoids. Single-cell trajectories for control (upper) and cancer (lower) organoid datasets predicted by Monocle3 and visualized by uniform manifold approximation and projection (UMAP) plots colored by (**a**) Seurat clusters and (**b**) pseudotime, in a differentiation gradient from purple to yellow using the clusters with high StemID scores as root nodes. (**c**) Selected markers for progenitor, stem, and differentiated cells plotted in pseudotime for the control (left) and cancer (right) datasets.

To investigate changes in gene expression during the differentiation of stem/progenitor cells in the control and cancer organoids, trajectory analysis of the Seurat objects was performed using the Monocle3 package (Fig. 3a). The clusters with the highest StemID values were denoted the root nodes and the pseudotime and trajectory paths were estimated (Fig. 3b). Selected markers for stem, proliferative, and differentiated cells were plotted in pseudotime for the control and cancer datasets (left and right, respectively, in Fig. 3c). In both control and cancer organoids, the expression of Hopx and the suprabasal keratinocyte markers Krt13, Krt4, and Krt6a increased with pseudotime while Lgr6-expressing cells were enriched in undifferentiated cells. The expression of Krt8 increased with pseudotime in the cancer organoids but was high in both stem-like and differentiated cells in the control organoids. Furthermore, the expression of Trp63 decreased gradually over pseudotime in cancer organoids but peaked in the middle of the trajectory in control organoids. The expression of Mki67 increased over pseudotime in the control organoids but peaked in the middle of the trajectory in the cancer organoids.

### Integration of control and cancer organoid datasets

**Figure 4:**
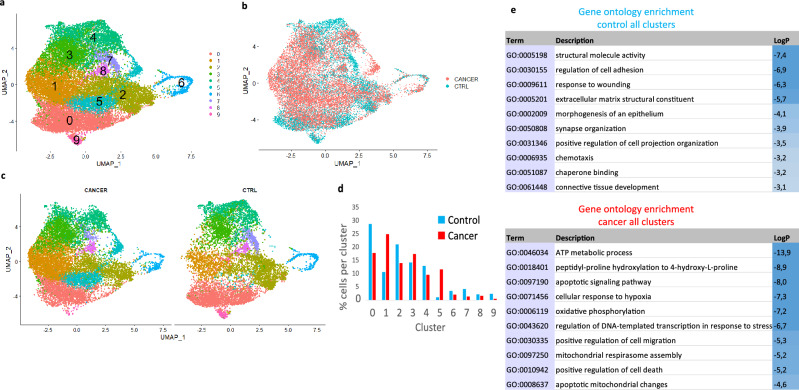
Integration of control and cancer data sets. Uniform manifold approximation and projection (UMAP) plot of the integrated Seurat object with cells labeled based on (**a**) Seurat clusters and (**b**) their origin [control (blue) or cancer (pink)]. (**c**) Integrated object split into separate UMAP plots for control and cancer cells. (**d**) Bar chart depicting the percentage of total cells contributing to each cluster [control (blue bars) and cancer (red bars) cells]. (**e**) Top 10 enriched Gene Ontology biological process terms in all clusters of control organoids when compared with cancer organoids (upper table) and vice versa (lower table).

For direct comparison in the same plot, the two datasets were integrated using reciprocal principal component analysis (RPCA) in Seurat, which generated an integrated Seurat object with nine clusters (Fig. 4a–c). The contribution of cells from each sample is shown in the bar graph in Fig. 4d. To analyze the broad differences between control and cancer organoids, the genes that were the most significantly altered between the control and cancer organoids in all clusters were determined using Seurat. These genes were subjected to gene ontology (GO) enrichment analysis (Fig. 4e). The analysis revealed the regulation of cell adhesion, response to wounding, extracellular matrix, positive regulation of cell projection organization, and chemotaxis among the most enriched terms in the control organoids while terms such as ATP metabolism, apoptosis, response to hypoxia, and cell migration were enriched in the cancer organoids.

### Characteristics of stem-like cells in control and cancer organoids

**Figure 5:**
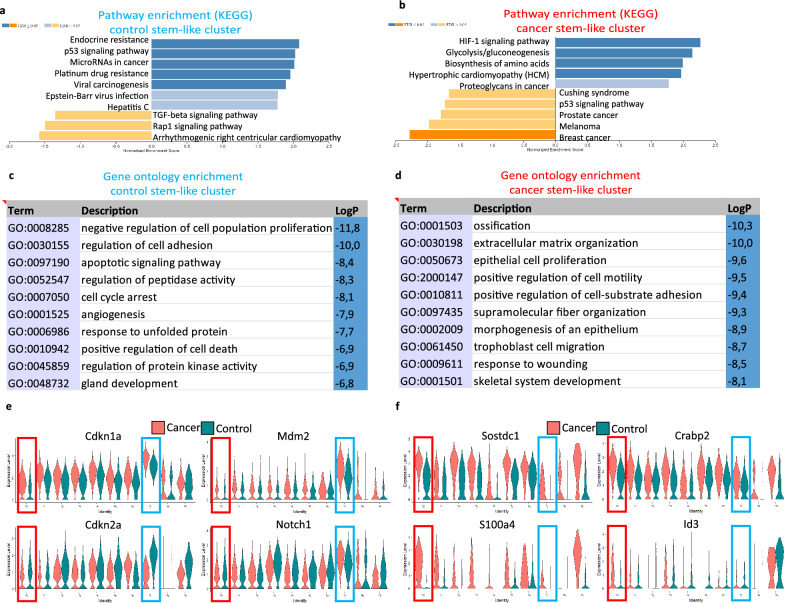
Kyoto Encyclopedia of Genes and Genomes (KEGG) pathway enrichment, Gene Ontology (GO) enrichment, and gene expression analyses in control and cancer stem-like cell clusters of the integrated object. (**a**) Bar graph showing upregulated (blue) and downregulated (yellow/orange) KEGG pathways in the control stem-like cell cluster (cluster 7) compared to the cancer stem-like cell cluster (cluster 0). (**b**) Bar graph showing upregulated (blue) and downregulated (yellow/orange) KEGG pathways in the cancer stem-like cell cluster (cluster 0) compared to the control stem-like cell cluster (cluster 7). Dark blue and orange bars in (**a**) and (**b**) indicate false discovery rate (FDR) ≤ 0.05, while light blue and yellow bars indicate FDR > 0.05. (**c**) Top 10 enriched GO biological process terms in the control stem-like cell cluster. (**d**) Top 10 enriched GO biological process terms in the cancer stem-like cell cluster. (**e**) Violin plots of selected genes upregulated in the control stem-like cell cluster. (**f**) Violin plots of selected genes upregulated in the cancer stem-like cell cluster. Red (cancer) and blue (control) rectangles highlight the stem-like clusters in (**e**) and (**f**). KEGG is developed by Kanehisa Laboratories (http://www.kegg.jp/kegg/kegg1.html).

Comparing the expression of markers for the clusters with the highest StemID values in the control and cancer data sets with their expression in the integrated object, we found that cell populations with stem-like features from the two datasets were clustered separately. Stem-like cells from the control and cancer organoids clustered in clusters 7 and 0, respectively, of the integrated object (Supplementary Fig. 2). To analyze the differences between stem-like cells from cancer and control organoids, the genes that were most significantly altered between the two clusters were identified using Seurat. These genes were subjected to enrichment analysis to identify the pathways and biological processes that were the most significantly altered between the two clusters. Kyoto Encyclopedia of Genes and Genomes (KEGG) pathway analysis revealed that the p53 signaling pathway was upregulated in the control cells (Fig. 5a), whereas hypoxia-related pathways, such as HIF-1 signaling and glycolysis, were upregulated in the cancer cells (Fig. 5b). GO enrichment analysis revealed that apoptotic signaling, cell cycle arrest, and negative regulation of cell population proliferation were among the most significantly enriched terms in the control stem-like cell cluster. Extracellular matrix organization, epithelial cell proliferation, and positive regulation of cell motility were enriched terms in the cancer stem-like cell cluster (Fig. 5c,d). Several cell cycle inhibitors, including Cdkn1a, Cdkn2a, Mdm2, Trp53inp1, Ccng, and Notch1—a known tumor suppressor in head and neck squamous cell carcinoma (HNSCC)^12^—were upregulated in the control stem-like cell cluster but not in the cancer stem-like cell cluster (Fig. 5e; Supplementary Table 1). The expression of known cancer-related genes such as Sostdc1, S100a4, Crabp2, and Id3 was significantly upregulated in the cancer stem-like cell cluster (Fig. 5f; Supplementary Table 2).

## Discussion

To improve the treatment of heterogeneous malignancies, such as tongue carcinoma, tumor cells—particularly cell populations with stem cell properties—must be characterized at the single-cell level. To the best of our knowledge, there has been only one study on single-cell RNA-sequence analysis of 4-NQO-induced tongue cancer tissue in mice^13^. The authors reported that the most significantly enriched pathway in cancer-related clusters was the Myc targets regulatory pathway. Some studies performed bulk RNA sequencing of 4-NQO-induced tongue cancers. GO enrichment and pathway enrichment analyses revealed that angiogenesis, proliferation, and migration pathways were upregulated and that the citric acid cycle was downregulated in cancer tissue^14,15^.

The 4-NQO-induced tumor tissue is heterogeneous and comprises both non-cancerous and cancerous regions; therefore, it is difficult to interpret the analysis of gene expression of cells isolated directly from the tissues. Additionally, this analysis may fail to detect rare stem cell and progenitor populations. We therefore chose to enrich cancer and normal progenitor cells by subjecting them to 3D organoid culture before performing scRNA-seq analysis. Some small, round organoids were observed in the culture of cells isolated from 4-NQO treated mice. However, most lingual organoids exhibited distinct characteristics including accelerated growth and the formation of bud-like proliferating structures. We previously demonstrated that organoids are generally generated by a single cell^5^. Irregularly shaped organoids should be derived from single immature cancerous cells with stem-like properties. The cancer organoids mimicked the histological features of tongue cancer tissue as evidenced by the expression patterns of markers such as Ki-67 and Krt14.

scRNA-seq analysis indicated that, consistent with organoid growth patterns, the proliferation of stem-like cells in the control organoids was tightly regulated when compared with that of stem-like cells in the cancer organoids. The p53 signaling pathway was activated and the expression of genes involved in adhesion, apoptosis, and negative control of cell division was upregulated in the control organoids. The expression of tumor suppressor genes, such as Cdkn2a and Notch1, was downregulated in stem-like cancer cells compared to control stem-like cells. This was consistent with the situation in human oral cancer where loss or mutation of these genes are frequent^12,16^.

Cancer organoid stem-like cells showed enrichment in proliferation, motility, and migration gene sets and upregulation of pathways such as HIF-1 signaling and glycolysis. These pathways are activated by hypoxia, which is a common feature of many solid tumors including oral squamous cell carcinoma^17^. The active uptake of glucose and glycolysis under aerobic conditions (the Warburg effect) supplies energy and metabolites necessary to support the increased proliferation of cancer cells in various tissues and may be the mechanism underlying the uncontrolled proliferation of cancer organoids^18^. These findings were consistent with those of previous studies, which reported the activation of angiogenesis and hypoxia pathways in 4-NQO-induced murine tongue cancer models^14,15^. Thus, these findings support the hypothesis that organoids can be used as in vitro models for tongue cancer.

Several novel genes including Sostdc1, S100a4, Crabp2, and Id3 were identified among the top upregulated genes in the cancer stem-like cell population. Sostdc1 is a BMP/Wnt antagonist that often functions as a tumor suppressor, but was reported to promote invasion and liver metastasis in the case of colorectal cancer^19^. S100a4 is an epithelial-mesenchymal transition mediator associated with the maintenance of cancer-initiating cells in HNSCC^20^. Aberrant expression of Crabp2 is correlated with oncogenesis in lung and liver cancers^21,22^. Id3, often in concert with Id1, is essential for the self-renewal of tumor-initiating cells in several malignancies including glioma, breast cancer, and colon cancer^23–25^. Future studies should focus on the expression of these genes in murine lingual cancer tissue and human primary cancer samples and examine the effect of a targeted knockdown of these genes on the proliferation of cancer organoids.

To the best of our knowledge, this is the first study to analyze the gene expression profiles of control and cancer organoids derived from the mouse lingual epithelium at the single-cell level in a comparative manner. Future work should study gene expression patterns of organoids from various stages of cancer progression. This study analyzed the organoids isolated at one time point from one control and one 4-NQO-treated mouse. However, the organoid culture of lingual epithelial cells recapitulated the phenotypic and gene expression characteristics of healthy and malignant lingual epithelia. Thus, organoid culture can be used as a model to further characterize cancer cells and screen anti-cancer drugs in vitro. The findings of this study will contribute to elucidating the characteristics of heterogeneous cell populations with stem cell properties in tongue tumors, thereby enabling the development of novel molecular-targeted cancer therapies for tongue cancers based on specific genes expressed in cancer stem-like cell populations.

## Methods

### Mice

Mice were bred and maintained at the Kansai Medical University Research Animal Facility according to the Kansai Medical University guidelines. B6;129S-*Sox2*^*tm2Hoch*^/J (Sox2-GFP; JAX#017592) mice were purchased from Jackson Laboratories (Sacramento, CA).

### Ethics statement

The animal experiments were approved by the Kansai Medical University Animal Experiment Committee (approval numbers: 20-110, 20-128) and performed according to the Guidelines for Animal Experimentation, Kansai Medical University, and the ARRIVE guidelines (https://arriveguidelines.org).

### Induction of tongue tumors

A stock 4-NQO solution was diluted in drinking water to a concentration of 100 µg/mL and administered to Sox2-GFP mice for 16 weeks. The mice were administered with plain distilled water for 8 additional weeks and then sacrificed. The tongue was harvested for immunohistochemical analysis and lingual epithelial cell culture.

### Establishment of mouse lingual organoids

The tongue was excised from the euthanized control and 4-NQO-treated mice and incubated with dispase (50 U/mL) for 10 min at 37 °C. The epithelial tissue was peeled off the tongue, minced into small pieces, and incubated in TrypLE Express (Thermo Fisher Scientific, Waltham, MA) for 30 min at 37 °C with pipetting every 10 min. The cell suspension was then passed through a 70 µm filter into 5 mL of culture medium. The filtrate was centrifuged at 440*g* for 5 min. The supernatant was removed such that 3 mL remained and the pellet was resuspended in the remaining supernatant. The cell suspension was passed through a 40 µm filter into a fluorescence-activated cell sorting tube. The filtrate was centrifuged for 5 min and the supernatant was discarded. The pellet was resuspended in phosphate-buffered saline (PBS) supplemented with 10% fetal bovine serum and incubated with 2.5 µg/mL of 7-amino-actinomycin D (7AAD) viability staining solution (Thermo Fisher Scientific) for 5 min to label the non-viable cells. The samples were then subjected to sorting in a SH800S cell sorter (SONY, Tokyo, Japan). The 7AAD^−^ GFP^+^ cells were sorted and collected into one well of a 96-well plate. The cells were then centrifuged at 120*g* for 5 min. The supernatant was removed such that only 10 µL remained. The cell pellet was then resuspended in 90 µL Matrigel on ice and seeded in a collagen-coated plate. The Matrigel was allowed to polymerize for 5 min. The cells were cultured in an organoid culture medium [Advanced Dulbecco’s modified Eagle medium/F12 supplemented with penicillin/streptomycin, HEPES (10 mM), Glutamax, N2, B27, *N*-acetylcysteine (1 µM), epithelial growth factor (50 ng/mL), noggin (100 ng/mL), and R-spondin1 (1 µg/mL)]. ROCK inhibitor (Y-27632; 10 mM) was added to the medium for the first 3–4 days after plating but was not supplemented during subsequent changes of the medium. After a few days, organoids were visible in the Matrigel. The organoids were dispersed into single cells and replated after they reached a large size (days 7–14 post seeding).

### Immunohistochemistry

Tissues and organoids were fixed in 4% paraformaldehyde, frozen in OCT compound, and sectioned into 5-µm thick sections using a microtome. The sections were transferred to slides, dried, washed in PBS, blocked with 3% bovine serum albumin (BSA)/0.1% Triton-X/PBS for 1 h, incubated with the primary antibodies overnight, washed in PBS, and incubated with the secondary antibodies. The nuclei were stained with Hoechst33342 (1 µg/mL; Thermo Fisher Scientific; H3570). Antigen retrieval using citric acid treatment at 121 °C for 20 min was performed before incubation with Ki67, Sox2, and p63 antibodies.

### Antibodies

Tissues and organoids were subjected to immunostaining using the anti-Ki67 (1:1000; Thermo Fisher Scientific; 14-5698), anti-Sox2 (1:200; Abcam, Cambridge, UK; ab97959), anti-p63 (1:200; Santa Cruz Biotechnology, Dallas, TX; sc8344), anti-Krt14 (1:100; BioLegend, San Diego, CA; 905304), and anti-Krt8 (1:100; DSHB, University of Iowa, Iowa City, IA; TROMA-I) primary antibodies and Alexa 594-labeled and Alexa 647-labeled secondary antibodies (1:200; Thermo Fisher Scientific).

### Microscopy

The frozen sections were imaged using a FV3000 confocal laser scanning microscope (Olympus Corporation, Tokyo, Japan). The images were analyzed using the software ImageJ (National Institutes of Health, Bethesda, MD). Live images of organoids in culture were captured using a BZ-X710 All-in-One fluorescence microscope (Keyence Corporation, Osaka, Japan).

### scRNA-seq

The control and cancer organoids were enzymatically digested, sorted into single cells with an SH800 cell sorter (SONY), and suspended in PBS containing 2% BSA. The dead cells were excluded by staining the samples with 7AAD, which allowed the sorting of only live (7AAD^−^) cells. The cells were then processed using the Chromium Controller (10 × Genomics, Pleasanton, CA), Chromium Next GEM Single Cell 3′ GEM, Library and Gel Bead Kit v3 (10 × Genomics; PN-1000128), and MGIEasy Universal Library Conversion Kit (App-A) (MGI, Shenzhen, China) following the manufacturer’s instructions. The library was sequenced using DNBSEQ-G400 (MGI).

### Downstream analysis of 3′-scRNA-seq data

The scRNA-seq output data were processed with the Cell Ranger pipeline (10 × Genomics) against the mouse reference (mm10) datasets. The gene-barcode matrices were analyzed and visualized using the Seurat R package (version 4.0.1)^26^. Genes expressed in less than 3 cells and cells containing less than 200 genes were filtered out. To remove potential doublets and low-quality cells, cells with unique gene counts of more than 6500 or a mitochondrial gene percentage greater than 10% were discarded. After filtration, the control and cancer organoid datasets contained information on 17,642 genes expressed in 9164 cells and 17,134 genes expressed in 12,378 cells, respectively. The expression of each gene was normalized, and the data were log-transformed. Principal component (PC) analysis was performed using highly variable genes. The first 40 PCs were used to generate cell clusters with the Louvain algorithm (14 and 10 clusters in the control and cancer datasets) at a resolution of 0.5. Nonlinear dimensional reduction and visualization were performed using the UMAP algorithm and the PCs selected above. The RaceID3 algorithm was used to identify stem cell-related clusters^27^. The datasets of cells with more than 2000 transcripts per cell were filtered. Mitochondrial and ribosomal genes were excluded. K-medoids cluster calculations were performed. The lineage tree was calculated using the StemID2 algorithm included in the RaceID3 R package. The number of significant links and the transcriptome entropy (reflecting the uniformity of the transcriptome) were calculated for each cluster. The StemID score for each cluster was determined by multiplying the link number and Δentropy. Marker gene expression was determined for the RaceID clusters. The expression of these markers in the RaceID object and the corresponding Seurat object was used to determine which Seurat cell cluster corresponded to each RaceID cluster. Trajectory/pseudotime analysis was performed using the Monocle3 algorithm^28^. To order the cells according to pseudotime, the clusters with the highest StemID scores were chosen as the root nodes. The control and cancer datasets were integrated into one dataset using the reciprocal PCA method in Seurat to allow direct comparisons.

### KEGG pathway and GO enrichment analyses

Differentially expressed genes between cancer stem-like cell and control stem-like cell clusters (for the integrated Seurat object) or those between the control/cancer stem-like cell clusters and other cell clusters (for the individual control and cancer Seurat objects) were identified using the Seurat FindMarkers function with default parameters. KEGG pathway enrichment analyses of these differentially expressed genes and the graphical representation were performed using the WEB-based GEne SeT AnaLysis Toolkit (http://www.webgestalt.org/)^29^. KEGG is developed by Kanehisa Laboratories (http://www.kegg.jp/kegg/kegg1.html). GO enrichment analysis was performed using the web-based Metascape database (https://metascape.org/)^30^.

## Supplementary Information

Supplementary Information.

## Data Availability

The datasets analyzed in this study are available from the corresponding author upon reasonable request.
