# Supplementary material for: Characterization of normal and cancer stem-like cell populations in murine lingual epithelial organoids using single-cell RNA sequencing
Source: Sci Rep. 2021 Nov 16;11:22329. doi: 10.1038/s41598-021-01783-5 (PMC8595654; doi:10.1038/s41598-021-01783-5)
Supplement: Supplementary file 1 — Supplementary Information. [file 41598_2021_1783_MOESM1_ESM.pdf]

Supplementary information

Supplementary figure legends

Supplementary Table 1. Top 100 genes upregulated in cluster 7 when compared with cluster 0 in the integrated Seurat object.

Supplementary Table 2. Top 100 genes upregulated in cluster 0 when compared with cluster 7 in the integrated Seurat object.

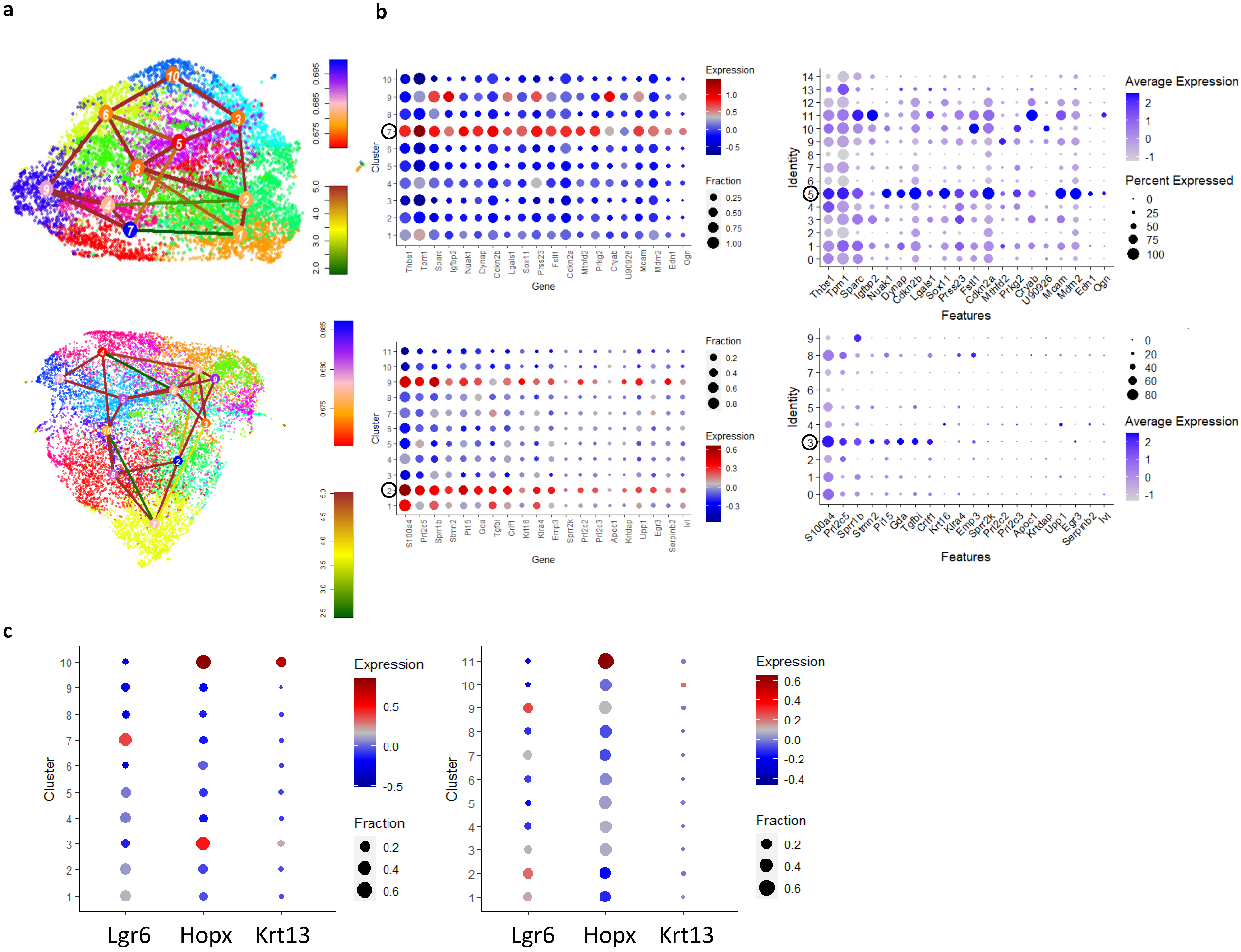

Supplementary Figure 1. (**a**) Uniform Manifold Approximation and Projection (UMAP) plots of control (upper) and cancer (lower) organoid RaceID datasets with superimposed lineage trees. (**b**) Dot plots of the top 20 marker genes for clusters with high StemID scores in the RaceID objects (left) and corresponding Seurat objects (right). Upper panel: control, lower panel: cancer. RaceID cluster 7 corresponds to Seurat cluster 5 for the control organoid data set, while RaceID cluster 2 corresponds to Seurat cluster 3 for the cancer organoid dataset. (c) Dot plots of selected genes in the control (left) and cancer (right) RaceID objects.

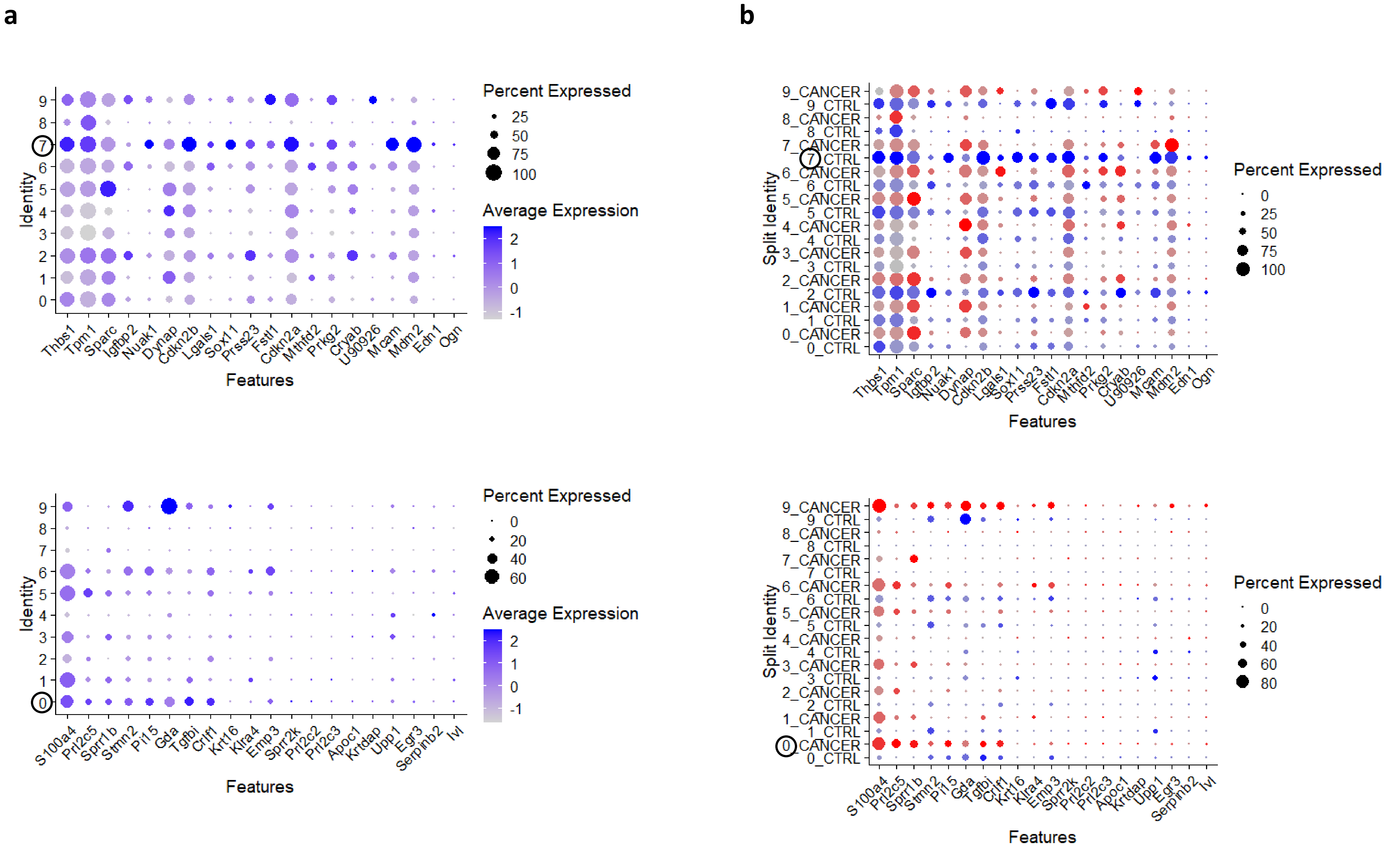

Supplementary Figure 2. Dot plots showing the expression of the top 20 markers in the stem cell clusters in the control (upper panel) and cancer (lower panel) objects in the integrated Seurat object without (**a**) or with (**b**) separation of the control and cancer objects. Control cluster 5 and cancer cluster 3 corresponds to cluster 7 and 0, respectively, in the integrated object.

Supplementary Table 1

gene	p_val	avg_log2FC	pct.1	pct.2	p_val_adj
Cdkn1a	2,34E-304	2,770536	1	0.952	4,67E-301
Cldn4	3,71E-282	2,767630	0.995	0.672	7,42E-279
Mdm2	3,28E-260	2,299156	0.989	0.732	6,55E-257
Gdf15	1,55E-281	1,996064	0.935	0.544	3,09E-278
Trp53inp1	2,04E-233	1,814635	0.968	0.606	4,08E-230
Sncg	9,88E-137	1,696742	0.998	0.987	1,98E-133
Ccng1	1,12E-262	1,688435	0.984	0.519	2,23E-259
Notch1	5,83E-157	1,443518	0.988	0.81	1,17E-153
Rps27l	4,51E-276	1,396875	1	0.963	9,02E-273
Zfp750	3,95E-270	1,381228	0.926	0.442	7,89E-267
Btg2	5,69E-142	1,370517	0.995	0.931	1,14E-138
9530053A07Rik	1,82E-257	1,255515	0.942	0.596	3,65E-254
Bax	8,16E-168	1,118611	0.981	0.872	1,63E-164
Krt8	6,68E-94	1,112708	0.979	0.901	1,34E-90
Krt18	2,03E-111	1,101930	0.993	0.901	4,07E-108
Fgfbp1	1,22E-168	1,101181	0.949	0.545	2,44E-165
Ppbp	6,38E-88	1,100529	0.85	0.612	1,28E-84
Jund	1,91E-131	1,079910	1	0.992	3,81E-128
Ptp4a3	1,88E-152	1,077122	0.975	0.72	3,76E-149
Tubb4b	2,87E-115	1,064924	0.989	0.885	5,73E-112
Ly6d	0,000515666	1,021791	0.549	0.493	1
Pkp1	4,16E-117	1,005449	0.977	0.881	8,31E-114
mt-Cytb	2,12E-148	0,990866	0.989	0.97	4,25E-145
Ces2e	4,35E-205	0,990113	0.933	0.617	8,69E-202
Pmaip1	2,09E-145	0,988161	0.905	0.492	4,19E-142
Psrc1	8,24E-161	0,987944	0.88	0.541	1,65E-157
Dcxr	2,87E-117	0,985828	0.993	0.907	5,73E-114
Nectin4	1,44E-180	0,965481	0.903	0.617	2,88E-177
Gabrp	2,76E-145	0,960147	0.848	0.583	5,52E-142
Atg9b	1,74E-89	0,943166	0.824	0.642	3,47E-86
Siva1	9,97E-126	0,920861	0.998	0.935	1,99E-122
Fosb	5,23E-52	0,876238	0.949	0.864	1,05E-48
Hist1h1c	2,08E-103	0,872742	0.905	0.742	4,17E-100
Cox6b2	4,73E-128	0,865943	0.727	0.256	9,46E-125
Mcam	1,39E-120	0,859410	0.776	0.242	2,78E-117
Cryab	6,20E-45	0,859297	0.808	0.668	1,24E-41
Bcl2l11	8,79E-112	0,854910	0.914	0.586	1,76E-108
Tnfsf9	1,08E-150	0,827513	0.935	0.662	2,16E-147
Net1	3,68E-64	0,819738	0.986	0.864	7,37E-61
Ly6e	6,71E-118	0,800575	0.998	0.978	1,34E-114
Dusp1	1,14E-53	0,789082	0.975	0.912	2,28E-50
Ckmt1	1,95E-87	0,778872	0.869	0.625	3,89E-84
Lgals1	0,650384741	0,744070	0.536	0.6	1
Bst2	4,70E-101	0,740072	0.862	0.707	9,40E-98
Tob1	1,48E-113	0,725308	0.951	0.748	2,97E-110
Tacstd2	1,02E-10	0,722017	0.977	0.907	2,03E-07
S100a14	8,85E-59	0,715534	1	0.999	1,77E-55

Ces2g	1,92E-178	0,710535	0.896	0.544	3,84E-175
mt-Nd4	9,22E-86	0,701311	0.996	0.98	1,84E-82
Anxa1	0,008048954	0,697243	0.996	0.993	1
Robo3	1,30E-172	0,696769	0.832	0.479	2,61E-169
Ddit4l	1,97E-54	0,696739	0.695	0.323	3,95E-51
Sat1	3,37E-48	0,692864	0.991	0.969	6,73E-45
Gsto1	4,03E-50	0,692366	0.998	0.984	8,06E-47
Areg	2,79E-120	0,690617	0.859	0.62	5,59E-117
Rab25	1,77E-63	0,685216	0.748	0.364	3,55E-60
Cldn3	8,31E-10	0,674120	0.577	0.516	1,66E-06
Atf3	1,30E-48	0,672581	0.966	0.915	2,61E-45
Tmprss11e	1,96E-163	0,671866	0.808	0.522	3,92E-160
Ppp1r15a	2,57E-83	0,667179	0.947	0.758	5,13E-80
Hspa1b	1,14E-59	0,658279	0.951	0.749	2,27E-56
Hist1h2bc	8,01E-90	0,653681	0.885	0.716	1,60E-86
Nudcd2	2,82E-71	0,650049	0.869	0.564	5,64E-68
Ddit4	4,13E-50	0,643897	0.942	0.754	8,25E-47
Spint1	1,37E-49	0,630312	0.656	0.289	2,74E-46
Nuak1	2,01E-31	0,623065	0.506	0.197	4,01E-28
Dynap	5,19E-11	0,609017	0.961	0.899	1,04E-07
Mcl1	3,19E-46	0,607318	0.958	0.879	6,38E-43
Ccnd1	1,03E-15	0,606538	0.956	0.853	2,06E-12
Fos	8,57E-09	0,601583	0.958	0.941	1,71E-05
Cenpm	1,24E-149	0,597638	0.753	0.19	2,47E-146
Ly6a	3,74E-12	0,594352	0.924	0.786	7,49E-09
mt-Nd3	1,07E-48	0,584735	0.954	0.735	2,14E-45
Prrg4	6,90E-40	0,576073	0.721	0.507	1,38E-36
Ecm1	0,000209797	0,565671	0.975	0.964	0,419594774
Fbxw9	9,03E-61	0,564767	0.82	0.612	1,81E-57
Rgs17	1,49E-29	0,562471	0.561	0.183	2,98E-26
Taldo1	1,51E-39	0,551217	0.965	0.87	3,03E-36
Endod1	6,51E-41	0,548004	0.847	0.743	1,30E-37
Dyrk3	6,32E-104	0,544213	0.884	0.646	1,26E-100
Cpne2	1,74E-37	0,539527	0.552	0.138	3,47E-34
Cdkn2a	9,75E-51	0,535928	0.901	0.721	1,95E-47
Serpine2	1,06E-54	0,531747	0.981	0.91	2,11E-51
Hspb1	3,20E-21	0,525216	1	0.999	6,41E-18
Ftl1	2,41E-59	0,525138	1	1	4,81E-56
Notch3	8,97E-07	0,524220	0.517	0.396	0,001793251
H2-K1	3,82E-39	0,523682	0.977	0.87	7,64E-36
Ptpn13	1,24E-47	0,513945	0.921	0.655	2,49E-44
Gas6	2,82E-15	0,509913	0.527	0.19	5,65E-12
mt-Atp6	2,23E-46	0,509591	0.995	0.995	4,46E-43
Plk2	1,87E-30	0,507099	0.935	0.9	3,75E-27
Hspa1a	1,67E-90	0,506225	0.855	0.448	3,33E-87
Avpi1	2,03E-34	0,502517	0.995	0.971	4,06E-31
Mybl1	4,09E-20	0,497584	0.519	0.278	8,18E-17
mt-Co3	5,54E-64	0,487456	0.998	0.999	1,11E-60
Hspb8	1,98E-51	0,482611	0.933	0.76	3,95E-48
Tpm1	1,23E-41	0,482481	1	0.999	2,46E-38

Cxcl1	4,72E-19	0,480041	0.813	0.578	9,44E-16
Cldn7	2,62E-07	0,476114	0.423	0.152	0,000524371
Hspa9	1,61E-35	0,469363	0.889	0.771	3,22E-32

Supplementary Table 2

gene	p_val	avg_log2FC	pct.1	pct.2	p_val_adj
Sostdc1	4,43E-247	2,387375	0.995	0.899	8,85E-244
S100a4	1,86E-222	2,363231	0.934	0.829	3,72E-219
Wfdc18	7,63E-150	1,924669	1	0.989	1,53E-146
Cbr2	1,10E-165	1,776824	0.932	0.783	2,20E-162
Prl2c5	3,51E-107	1,728556	0.798	0.75	7,02E-104
Rbp1	2,43E-211	1,707190	1	0.951	4,86E-208
Lgals7	1,16E-194	1,580235	1	0.977	2,32E-191
Lcn2	1,14E-78	1,482994	0.842	0.566	2,28E-75
Crabp2	4,65E-132	1,382736	0.992	0.945	9,30E-129
Ndufa4l2	6,50E-81	1,361223	0.906	0.882	1,30E-77
2610528A11Rik	2,30E-66	1,206832	0.829	0.804	4,61E-63
Tmem176a	3,44E-193	1,200377	1	0.989	6,87E-190
Krt14	7,20E-166	1,147948	1	0.991	1,44E-162
Pgf	1,07E-157	1,076795	0.908	0.806	2,13E-154
Fabp5	1,18E-64	1,064522	0.777	0.591	2,36E-61
Id3	1,31E-97	1,057633	0.798	0.344	2,62E-94
Hif1a	4,96E-129	1,055590	0.958	0.795	9,92E-126
Defb1	2,76E-113	1,041730	0.808	0.728	5,53E-110
Krt17	6,50E-125	1,021067	1	0.998	1,30E-121
Itga6	6,36E-141	1,020027	0.962	0.797	1,27E-137
Tns4	3,35E-124	1,013758	0.976	0.765	6,71E-121
Cp	2,98E-98	1,009351	0.931	0.785	5,97E-95
Klk8	1,45E-141	0,987998	0.97	0.852	2,91E-138
Crip1	6,77E-76	0,978201	0.867	0.797	1,35E-72
Golim4	4,38E-125	0,974736	0.994	0.979	8,76E-122
Sgk1	1,39E-96	0,972217	0.913	0.656	2,79E-93
Stra6	1,51E-103	0,964384	0.843	0.524	3,03E-100
Trp63	3,85E-113	0,963742	0.83	0.448	7,69E-110
Igfbp7	4,00E-97	0,958897	0.988	0.852	7,99E-94
Enpp1	8,12E-93	0,954819	0.827	0.709	1,62E-89
Tagln2	4,41E-125	0,951956	0.998	0.988	8,82E-122
Ucp2	2,59E-93	0,943787	0.794	0.494	5,18E-90
Stmn2	1,18E-13	0,938207	0.541	0.594	2,36E-10
Cav1	2,88E-106	0,920255	0.963	0.884	5,76E-103
Mgp	0,135230365	0,919972	0.464	0.601	1
4930523C07Rik	1,78E-98	0,882987	0.898	0.776	3,55E-95
Krt5	4,03E-120	0,859438	1	0.998	8,06E-117
Pglyrp1	5,16E-38	0,829077	0.898	0.859	1,03E-34
Nlrp10	6,56E-86	0,812628	0.959	0.903	1,31E-82
Sparc	9,47E-74	0,810317	0.994	0.949	1,89E-70
S100a10	6,91E-144	0,806966	1	0.996	1,38E-140
Cebpd	6,66E-22	0,796432	0.732	0.824	1,33E-18
Plec	2,06E-84	0,793668	0.814	0.554	4,12E-81
Sprr1a	1,73E-14	0,777241	0.869	0.898	3,47E-11
Tnfaip2	7,66E-62	0,775376	0.62	0.386	1,53E-58
Igfbp3	5,52E-54	0,771358	0.797	0.54	1,10E-50
Id1	3,45E-32	0,765363	0.804	0.725	6,91E-29

Efemp1	7,15E-70	0,762482	0.801	0.515	1,43E-66
S100a6	1,87E-88	0,753406	1	1	3,74E-85
Ndrg1	3,10E-52	0,751486	0.97	0.944	6,20E-49
Slc15a2	2,41E-71	0,723823	0.8	0.748	4,81E-68
Tmem176b	1,23E-95	0,716492	1	0.995	2,46E-92
Ctgf	1,03E-33	0,713689	0.819	0.66	2,07E-30
Car12	1,45E-53	0,710657	0.744	0.725	2,90E-50
Actb	2,33E-84	0,709100	1	1	4,66E-81
Cavin3	4,45E-70	0,702263	0.908	0.771	8,90E-67
Cyr61	1,09E-50	0,699950	0.863	0.598	2,19E-47
Dag1	1,46E-64	0,699525	0.964	0.919	2,92E-61
Gda	1,35E-67	0,698582	0.593	0.314	2,70E-64
Csrp1	2,16E-55	0,698552	0.928	0.884	4,33E-52
Clu	2,81E-83	0,696456	0.886	0.547	5,62E-80
Calm1	2,64E-65	0,689992	0.981	0.931	5,28E-62
Malat1	2,70E-25	0,686900	0.998	0.998	5,39E-22
Tubb5	7,13E-80	0,685919	0.995	0.952	1,43E-76
Vsnl1	1,98E-56	0,685513	0.682	0.436	3,96E-53
Gpx2	3,97E-55	0,680201	0.904	0.905	7,94E-52
Serpinb5	3,57E-64	0,675567	0.96	0.942	7,14E-61
Cavin1	1,86E-64	0,674863	0.841	0.66	3,71E-61
Prdx6	2,06E-87	0,674587	0.992	0.965	4,12E-84
Mt1	1,83E-44	0,666930	0.968	0.949	3,67E-41
Vwa1	6,63E-50	0,663095	0.776	0.755	1,33E-46
Gja1	8,85E-45	0,657974	0.892	0.869	1,77E-41
Bzw2	4,41E-62	0,649735	0.904	0.799	8,82E-59
Pgk1	3,07E-89	0,636850	1	0.998	6,13E-86
Lamb3	4,29E-68	0,636544	0.929	0.716	8,58E-65
Snai2	3,95E-43	0,631253	0.896	0.825	7,90E-40
4631405K08Rik	1,95E-55	0,617270	0.891	0.804	3,89E-52
Ehf	9,85E-55	0,613805	0.967	0.959	1,97E-51
Sorbs2	1,60E-48	0,609388	0.601	0.31	3,20E-45
Glul	1,31E-31	0,606229	0.926	0.875	2,61E-28
Arhgdib	2,77E-37	0,604768	0.874	0.825	5,53E-34
Dram1	6,27E-93	0,601208	0.579	0.201	1,25E-89
Crlf1	3,01E-161	0,598349	0.668	0.12	6,01E-158
Tgfbi	9,29E-41	0,593716	0.507	0.243	1,86E-37
Hlf	1,83E-20	0,590498	0.783	0.799	3,66E-17
Anxa2	2,98E-53	0,590367	0.999	0.995	5,97E-50
Zfp36l2	4,66E-45	0,588861	0.786	0.57	9,33E-42
Pi15	2,07E-33	0,584085	0.686	0.728	4,15E-30
Tspo	3,91E-51	0,573011	0.972	0.958	7,82E-48
Rbbp8	1,69E-65	0,571301	0.617	0.296	3,38E-62
Pls3	8,43E-46	0,567762	0.874	0.771	1,69E-42
Tmsb10	1,37E-54	0,564331	0.997	0.956	2,73E-51
Has2	1,63E-39	0,558875	0.468	0.238	3,26E-36
Hnrnpa3	4,49E-54	0,557918	0.941	0.903	8,98E-51
Dst	2,30E-45	0,553209	0.863	0.667	4,60E-42
Higd1a	7,62E-31	0,552354	0.969	0.954	1,52E-27
Fth1	2,82E-77	0,551775	1	1	5,64E-74

Sprr2a3	2,96E-31	0,549058	0.438	0.741	5,92E-28
Slc39a4	9,66E-76	0,548483	0.771	0.732	1,93E-72
Ero1l	2,31E-39	0,547443	0.997	0.989	4,63E-36
